# Cloning of a Novel *vpr* Gene Encoding a Minor Fibrinolytic Enzyme from *Bacillus subtilis* SJ4 and the Properties of Vpr

**DOI:** 10.4014/jmb.2006.06014

**Published:** 2020-08-21

**Authors:** Zhuang Yao, Yu Meng, Huong Giang Le, Se Jin Lee, Hye Sung Jeon, Ji Yeon Yoo, Hyun-Jin Kim, Jeong Hwan Kim

**Affiliations:** 1 Division of Applied Life Science (BK2 Four), Graduate School, Gyeongsang National University, Jinju 52828, Republic of Korea; 2 Institute of Agriculture and Life Science, Gyeongsang National University, Jinju 588, Republic of Korea

**Keywords:** *vpr*, *aprE*, fibrinolytic activity, *Bacillus subtilis*

## Abstract

We have previously characterized AprESJ4, the major fibrinolytic enzyme from *Bacillus subtilis* SJ4 (Yao *et al*., 2019). During that study, we observed a 68 kDa protein with fibrinolytic activity. In this study, we cloned the gene (*vprSJ4*) encoding the 68 kDa protein, a mature Vpr and minor protease secreted by *Bacillus* species. *vprSJ4* encodes a preproenzyme consisting of 810 amino acids (aa) including signal sequence (28 aa) and prosequence (132 aa). The mature enzyme (650 aa) has a predicted molecular weight of 68,467.35. Unlike Vprs from other *B. subtilis* strains, VprSJ4 has 4 additional amino acids (DEFA) at the C-terminus. *vprSJ4* was overexpressed in *Escherichia coli*. PreproVprSJ4 was localized in inclusion bodies, and subjected to in vitro renaturation and purification by an affinity column. SDS-PAGE and western blot showed that autoprocessing of preproVprSJ4 occurred and 68 kDa and smaller proteins were produced. The optimum pH and temperature of the recombinant VprSJ4 were pH 7.0 and 40°C, respectively. Kinetic parameters of recombinant VprSJ4 were measured by using an artificial substrate, *N*-succinyl-ala-ala-pro-phe-*p*-nitroanilide. Coexpression of *vprSJ4* and *aprESJ4* using pHY300PLK increased the fibrinolytic activity a further 117% when compared with *aprESJ4* single expression using the same vector in *B. subtilis* WB600.

## Introduction

*Bacillus* species secrete several different proteases into culture medium at the end of log growth stage. Alkaline protease (Apr) and neutral protease (Npr) are the most important and major enzymes [1]. Other minor proteases include bacillopeptidase F, metallopeptidase, Vpr, Epr, and WprA [2]. Secreted proteases synthesized at the end of exponential growth phase help the host cells adapt to changed environments where nutrients, especially nitrogen sources, are limited [3]. However, the exact roles of each protease are still largely unknown. Some proteases possess strong fibrinolytic activities, and nattokinase, belonging to subtilisins (alkaline proteases), is the most well known example [4]. Bacillopeptidase F is another example and is also currently utilized as a thrombolytic agent together with nattokinase [5]. Nattokinase and bacillopeptidase F have been subjects of intensive study during last decades because they are considered promising alternatives for the medically important thromolytic agents such as t-PA (tissue plasminogen activator), urokinase, and streptokinase. Although these agents have been used to treat cardiovascular diseases caused by fibrin accumulation in the blood vessels, they have several shortcomings such as high cost, short in vivo half-lives, and internal bleeding [6]. *B. subtilis* and closely related *Bacillus* species are promising producers of more safe and economical fibrinolytic agents since they are considered as GRAS (generally recognized as safe).

In addition to providing peptides and amino acids for growth, proteases are responsible for other important functions. Subtilisin (AprE), Epr, and Vpr were shown to produce CSF (competence and sporulation factor), an extracellular signaling peptide, from its precursor [7]. AprE, WprA, and Vpr are responsible for the maturation of subtilin, a peptide antibiotic produced by *B. subtilis* [8]. More studies are necessary to discover the role of each protease on the growth of host cell during stationary phase.

Fermented foods are good sources for *Bacillus* sp. with strong fibrinolytic activities. A number of *Bacillus* sp. have been isolated from popular, traditional Korean fermented foods such as cheonggukjang, doenjang, jeotgal, and kimchi [9-12], and their AprE (subtilisin)-type proteases have been characterized most often. In this work, we cloned a *vpr* gene (*vprSJ4*) from *B. subtilis* SJ4, an isolate from saeu jeotgal (small salted shrimp), followed by fermentation at room temperature for several months [12]. The gene product, VprSJ4, showed fibrinolytic activity. *vprSJ4* was overexpressed in *E. coli*, the recombinant enzyme was purified, and the properties were studied. *vprSJ4* was also coexpressed with *aprESJ4* in *B. subtilis* WB600. The results will help us better understand the fibrinolytic system of *B. subtilis* SJ4 and find ways to use the strain in production of fermented foods or bioactive materials.

## Materials and Methods

### Bacterial Strains and Culture Conditions

*B. subtilis* SJ4 and *Escherichia coli* strains were grown in Luria-Bertani broth (LB, tryptone 10 g, yeast extract 5 g, NaCl 5 g, per liter, pH 7.0) at 37°C with aeration. Chromosomal DNA of *B. subtilis* SJ4 was prepared from culture grown overnight in LB. Culture was centrifuged (12,000 ×*g*, 5 min at 4°C), and cells were resuspended in 1 ml of lysis buffer (10 mM Tris-HCl, 10 mM EDTA, 100 mM NaCl, 2% SDS, and 400 μg/ml proteinase K (Takara, Japan, pH 8.0). After 30 min of incubation at 55°C, phenol and chloroform extraction was done followed by isopropanol precipitation [13].

### SDS-PAGE and Fibrin Zymography

*B. subtilis* SJ4 was grown in LB for 96 h, and the culture supernatant was prepared at 12 h intervals. Proteins were concentrated by trichloroacetic acid (TCA) precipitation. A 10% acrylamide gel (5% stacking gel) was used and 10 μg sample was loaded after being boiled for 3 min in SDS sample buffer. Fibrin zymography was done using a 10% gel containing fibrin, which was prepared by mixing fibrinogen (0.12%, w/v) and 100 μl of thrombin (10 NIH units/ml) with acrylamide solution. Culture supernatant (1 μg protein) was directly loaded without TCA precipitation, and electrophoresis was done at 10 mA in a cold room. After electrophoresis, the fibrin gel was soaked in 50 mM Tris-HCl buffer (pH 7.4) containing 2.5% Triton X-100 for 30 min at room temperature (RT) on a rotary shaker. The gel was washed with distilled water for 30 min to remove Triton X-100 and soaked in zymogram reaction buffer (30 mM Tris-HCl, pH 7.4, and 0.02% NaN3) for 12 h at 37°C. The gel was stained with Coomassie Blue R-250 for 1 h, and destained for 4 h [14].

### Cloning of vprSJ4

A primer pair was designed based on the published *vpr* gene sequence, vprSJ4F (5’-CGCGGATCCTTGAAA AAGGGGATCATTCGCTTTC-3’, BamHI site underlined) and vprSJ4R (5’-ACGCGAATTCGTCTTCAACAG TGAA AGGTTCTTCGGAC-3’, EcoRI site underlined) [15]. PCR was done using an MJ Mini Personal Thermal Cycler (BioRad, USA). The reaction mixture (50 μl) contained 1 μl of template DNA, 1 μl of each primer (10 μM), 1 μl of deoxynucleoside triphosphates (0.25 mM), and 0.5 μl of Ex *Taq* DNA polymerase (Takara). The amplification conditions were as follows: initial denaturation at 94°C for 5 min, 30 cycles of 94°C for 0.5 min, 69°C for 0.5 min, and 72°C for 2 min, and final extension at 72°C for 4 min. Amplified fragment was ligated with pGEM-T Easy Vector (Promega, USA), and the ligation mixture was introduced into *E. coli* DH5α competent cells by electroporation [16]. Plasmid DNA was prepared by using a plasmid DNA purification kit (iNtRon, Korea). Restriction enzyme digestion and agarose gel electrophoresis were done according to the published methods [17].

### Overexpression of *vprSJ4* in *E. coli* BL21 (DE3)

*vprSJ4* was amplified without its own signal sequence using the following primer pairs, pETvprSJ4F (5’-AGAGGATCCGATGGCTCCGGCTTCT-3’, BamHI site underlined) and pETvprSJ4R (5’-AGACTCGAGCGC GAATTCGTCTTC-3’, XhoI site underlined). The amplified fragment was inserted into pET26b(+) (Merck Millipore, Germany) after being digested with BamHI and XhoI. *E. coli* BL21 (DE3) competent cells were transformed with the ligation mixture by electroporation (200 Ω, 18 kV/cm) [16]. *E. coli* cells harboring pETvprSJ4 were grown in LB (250 ml) containing kanamycin (30 μg/ml) until the OD_600_ of culture reached 0.8. IPTG (isopropyl β-D-1-thiogalactopyranoside) was added to the final concentration of 1 mM, and the culture was incubated for 20 h at 30°C. After centrifugation at 4,000 ×*g* for 20 min at 4°C, the cell pellet was resuspended in 5 ml of phosphate-buffered saline (PBS, pH 7.2). Cells were then disrupted by sonication (5 cycles of 1 min sonication and 2 min cooling on ice) using a sonicator (UW 2070, Germany). Disrupted cells were centrifuged at 12,000 ×*g* for 15 min at 4°C. The supernatant (soluble fraction) and cell pellet (insoluble fraction) were obtained and used for experiments.

### In Vitro Renaturation and Purification of VprSJ4

Insoluble fraction as described above was dissolved in 6 M guanidine-HCl. After standing for 4 h at RT, the sample was dialyzed against an excess volume of Tris-HCl buffer (2 M, pH 7.0) containing reducing agents (5 mM cysteine and 1 mM cystine) for 12 h at 4°C. Then, the dialysate was centrifuged for 20 min at 12,000 ×*g* at 4°C to remove precipitate. Buffer exchange of the dialysate was done using binding buffer (20 mM sodium phosphate, 0.5 M NaCl, 10 mM imidazole, pH 7.4) and Amicon filters (MWCO 12,000, Millipore, USA). The dialyzed sample was then loaded onto a HiTrap IMAC FF Column (GE Healthcare, Sweden). VprSJ4 was eluted from the column by stepwise elution of buffer with increased imidazole concentration. The imidazole concentration was increased stepwise starting from 100 mM to 500 mM with 100 mM increments. Fractions were checked by SDS-PAGE. Protein concentration was measured by Bradford method using bovine serum albumin as a standard [18].

### Western Blot Analysis of VprSJ4

Eluents from the affinity column were separated by SDS-PAGE and proteins were transferred onto a PVDF membrane (Thermo Fisher, USA) by using a mini trans-blot electrophoretic transfer apparatus (BioRad). Then, membrane was incubated in blocking PBS buffer containing 3% skim milk at RT for 2 h with shaking, and incubated overnight at 4°C with anti-His antibodies (1/500 dilution, monoclonal antibody, mouse IgG1, Thermo Fisher). The membrane was incubated with the secondary antibody (1/3,000 dilution, anti-Mouse IgG (H+L) conjugated with alkaline phosphatase, Bio-Rad) at 37°C for 1 h with shaking. After washing with PBST (PBS containing 0.1% (v/v) Tween-20), alkaline phosphatase substrate (Bio-Rad) was added to develop color (RT, 5 min).

### Properties of Recombinant VprSJ4

The effect of pH on the fibrinolytic activity of VprSJ4 was examined by using different buffers (50 mM each): citrate-NaOH for pH 3-5, sodium phosphate for pH 6-8, and Tris-HCl for pH 9-11. VprSJ4 (1 µg) was resuspended in each buffer and incubated for 1 h at 40°C and the fibrinolytic activities were measured by the fibrin plate method [19]. For testing pH stability, VprSJ4 in each buffer was incubated at 40°C and the activities were measured at 1, 3, and 6 h. VprSJ4 in sodium phosphate buffer (pH 7) was incubated for 30 min at 37-60°C and the activities were measured to determine the optimum temperature. To test thermal stability, VprSJ4 in sodium phosphate buffer (pH 7) was incubated at 37-60°C and the activities were measured at 0.5, 1, 2, and 3 h. VprSJ4 in sodium phosphate buffer (pH 7) was exposed to 5 mM metal ion or 1 mM inhibitor for 30 min (40°C). The remaining activities were then measured.

### Amidolytic Activity and Enzyme Kinetics

*N*-Succinyl-ala-ala-pro-phe-*p*-nitroanilide (S7388, Sigma) was used as a substrate for amidolytic activity of VprSJ4. Fifty microliters of substrate (10 mM in sodium phosphate buffer, pH 7.0) was mixed with 10 μl of VprSJ4 (1 μg) and 440 μl of sodium phosphate buffer. The mixture was incubated at 40°C for 20 min, and 500 μl of citrate-NaOH buffer (pH 3.0) was added and put on ice immediately. The mixture was centrifuged at 12,000 ×*g* for 5 min and the absorbance of the supernatant was measured at 410 nm. The degree of hydrolysis was calculated from the absorbance value and the molar extinction coefficient value of *p*-nitroanilide (8,800 M^-1^ cm^-1^) [19]. Kinetic parameters of VprSJ4 were determined by measuring the release of *p*-nitroaniline from *N*-succinyl-ala-ala-pro-phe-*p*-nitroanilide in sodium phosphate buffer (100 mM, pH 7.0) containing 4% (v/v) DMSO at 37°C [19]. Vpr eluted at a concentration of 200 mM imidazole was further concentrated by using an ultra centrifugal filter (Amicon, USA, MWCO, 50 K). The concentrated Vpr was used for kinetic measurements. *V*_max_ and *K*_m_ were determined from measurements at different substrate concentrations ranging from 0.03 to 0.8 mM. *V*_max_ was converted to k_cat_ from the relationship *K*_cat_ = *V*_max_/[enzyme].

### Coexpression of *aprESJ4* and *vprSJ4* in *B. subtilis* WB600

*aprESJ4* and *vprSJ4* were amplified by PCR. Primers used were as follows: aprE-F (5’-ATCTAGACAAGAGAG CGATTGCGGCTGTG-3’, XbaI), aprE-R, (5’-AGGATCCTTCAGAGGGAGCCA CCCGTCG-3’, BamHI), vpr-F (5’-CGGATCCTACACAGGTTTATTCACTTA TAC-3 BamHI), and vpr-R (5’-GGAATTCGAAGATAAGCT CCGATCGTAT-3’ EcoRI). Restriction site is underlined. PCR was done as described above. *aprESJ4* (1.5 kb) and *vprSJ4* (2.7 kb) were digested separately. Then both genes were mixed and ligated with pHY300PLK cut with XbaI and EcoRI. The ligation mixture was introduced into *B. subtilis* WB600 competent cells by electroporation. *B. subtilis* WB600 was kindly provided by Dr. S. L. Wong (University of Calgary, Canada) [20]. Gene Pulser II (BioRad) was used and the conditions were 200 Ω, 25 μF and 2.1 kV/cm. After pulse, 1 ml of LB broth with 0.5 M sorbitol and 0.38 M mannitol was added and the cells were recovered for 3 h at 37°C with shaking.

*B. subtilis* WB600 TFs were grown in LB containing tetracycline (10 μg/ml) for 96 h. Aliquots of culture were taken at 12 h intervals, and culture supernatant was obtained. Fibrinolytic activities, SDS-PAGE, and fibrin zymography were done for the culture supernatants as described above.

## Results and Discussions

### Fibrinolytic Enzymes from *B. subtilis* SJ4

**Fig. 1:**
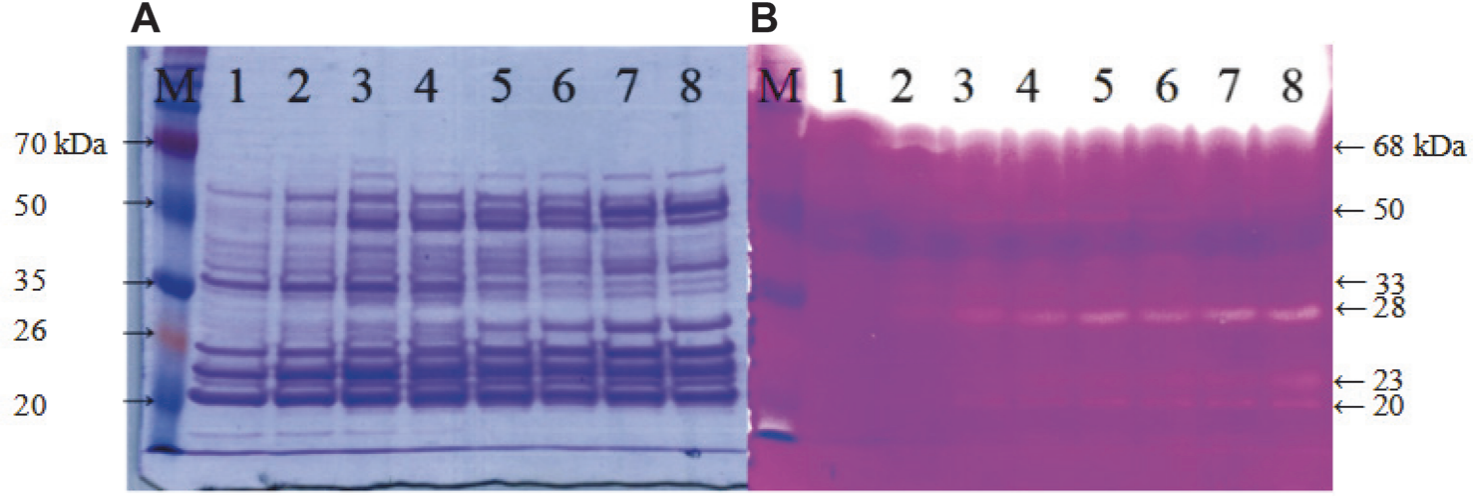
Coomassie Blue-stained gel (A) and fibrin zymogram (B) of supernatant from *B. subtilis* SJ4. M, Dokdo-marker (EBM-1032, Elpis-Biotech., Korea). *B. subtilis* SJ4 was grown in LB broth for 96 h at 37°C. 1, 12 h; 2, 24 h; 3, 36 h; 4, 48 h; 5. 60 h; 6, 72 h; 7, 84 h; 8, 96 h. 10 μg protein concentrated by TCA precipitation was used for SDS-PAGE, and 1 μg protein without TCA precipitation was loaded for fibrin zymography. 10% acrylamide gels were used with 5% stacking gels.

*B. subtilis* SJ4 was grown in LB for 96 h. Culture supernatants obtained at 12 h intervals were analyzed by SDS-PAGE and fibrin zymography (Fig. 1). As reported previously, 28 kDa protein is the mature AprESJ4, and the major fibrinolytic enzyme (Fig. 1B) [12]. The band intensity of AprESJ4 became stronger at 48 h and thereafter. Other proteins with fibrinolytic activity were observed on the zymogram, and they were 68, 50, 36, 23, and 20 kDa in size (Fig. 1B). These fibrinolytic proteins were also found at the corresponding positions on SDS-PAGE gel (Fig. 1A). The 68 kDa band in the zymogram was suspected to be Vpr considering the known size of the mature Vpr [2]. We decided to clone *vpr* gene from *B. subtilis* SJ4, and study the properties of Vpr. Moreover, *B. subtilis* SJ4 possesses significant salt tolerance, and can grow well in the presence of NaCl up to 15% (w/v) [12], which is a big advantage if *B. subtilis* SJ4 is used as a starter for jeotgal and other fermented foods with high salt content. Considering its high salt tolerance, GRAS status, and strong fibrinolytic activity, we believe that studies on other fibrinolytic enzymes from *B. subtilis* SJ4 are necessary.

### Analysis of Vpr

**Fig. 2:**
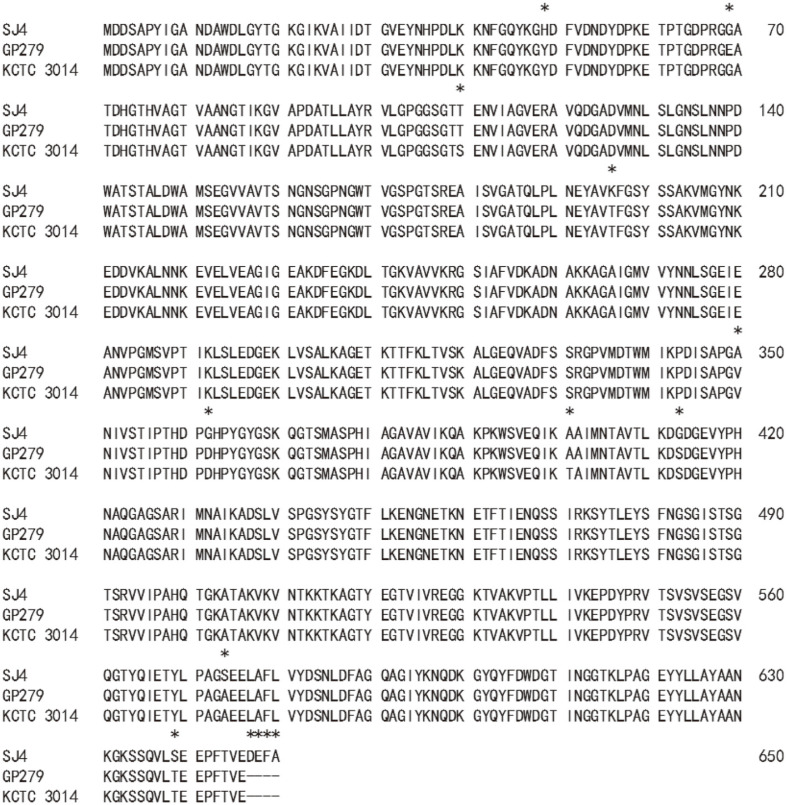
Alignment of amino acid sequence of mature VprSJ4 with homologous enzymes, Vpr from *B. subtili*s GP279 (M76590), and Vpr from *B. subtilis* KCTC 3014 (AY973268). Amino acids different from each other are marked with an asterisk above.

A 2.5 kb PCR fragment was ligated into pGEM-T easy vector. The recombinant plasmid, pGEMSJ4 (5.5 kb, Ap^r^), was sequenced (2,506 nucleotides, MN055601), and BLAST analysis located a *vpr* gene. The ORF was 2,433 bp in size (Fig. S1), encoding a protein of 810 amino acids. The molecular weight and pI were 85,955.70 Da and 6.14, respectively. The first 28 amino acids seemed to be a signal peptide as judged by the signalP 4.1 server (Technical University of Denmark). The next 132 amino acids were suspected to be a prosequence when compared with other Vpr enzymes [21], and the first amino acid of the mature enzyme was methionine at 161^st^. The pI and molecular mass of the mature enzyme were 5.25 and 68,467.35 Da, respectively. Although a 68 kDa band was not located on SDS-acrylamide gel (Fig. 1A), a strong fibrinolytic zone was observed at 68 kDa position on the fibrin zymogram just below the top strong hydrolytic zone (Fig. 1B). When the amino acid sequence of VprSJ4 was compared with those from other Vpr enzymes, high similarity was observed: 98.15% (638/650) with Vpr from *B. subtilis* GP279 (M76590) [21], and 98% (637/650) with Vpr from *B. subtilis* KCTC3014 (AY973268) [22]. Vpr from *B. subtilis* GP279 showed 99.5% identity with that from *B. subtilis* KCTC 3014, excepting a difference of 3 amino acids (69, 110. 401^th^). However, VprSJ4 differed from the Vpr from *B. subtilis* GP279 and *B. subtilis* KCTC3014 by 12 and 13 amino acids, respectively (Fig. 2). Unlike 2 other Vprs, VprSJ4 has an additional 4 amino acids (DEFA) at the C-terminus (Fig. 2), making VprSJ4 slightly larger than other Vprs. It will be interesting to study the effect of these 4 extra amino acids on the enzyme properties, including the C-terminus cleavage.

### Overexpression of *vprSJ4* in *E. coli* and Purification of Recombinant VprSJ4

VprSJ4 was overproduced in *E. coli* as a fusion protein containing 6 additional His (6 x His-tag) at the C-terminus, which facilitated the purification of recombinant VprSJ4 by affinity chromatography. When both soluble and insoluble fractions were analyzed by SDS-PAGE, a thick 86 kDa band was observed mainly from insoluble fraction (Fig. S2), indicating that VprSJ4 was not processed properly and aggregated as an inclusion body in *E. coli* [15]. The size matched that of preproenzyme (86 kDa).

**Fig. 3:**
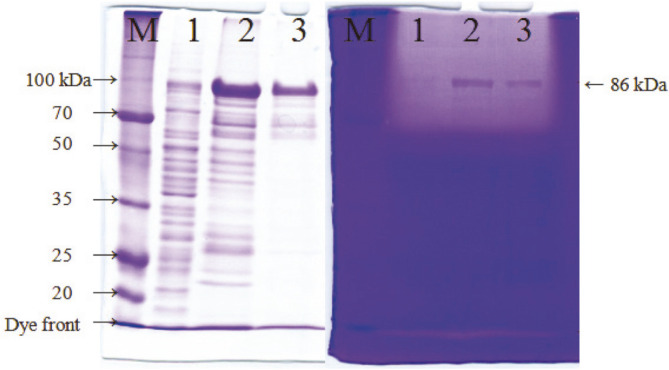
Purification of recombinant VprSJ4 from insoluble fraction. M, Dokdo-marker (EBM-1034, Elpisbio, Korea); 1, insoluble fraction; 2, eluent at 100 mM imidazole concentration; 3, eluent at 200 mM imidazole concentration.

VprSJ4 from insoluble fraction was purified by using a HiTrap IMAC FF Column after in vitro renaturation steps. PreproVprSJ4 was eluted from the column at 100 mM imidazole concentration together with other minor proteins. Additional preproVprSJ4 was eluted with a few other proteins at 200 mM imidazole concentration. PreproVprSJ4 was no longer eluted at 300 mM and higher imidazole concentrations (results not shown). Insoluble fraction and eluents at 100 and 200 mM imidazole concentration had fibrin degrading activities, shown as a big halo at the top of the fibrin gel (Fig. 3B). The results indicated that preproVprSJ4 was processed and some active forms were regenerated through in vitro renaturation processes. The active 68 kDa band was not located on the fibrin gel because the location was masked by the big halo at the top. However, a band of 68 kDa in size was observed in an SDS-gel (lanes 2 and 3, Fig. 3). Although other fibrinolytic bands were not located on the fibrin zymogram, it was still possible that smaller proteins were generated from preproVprSJ4 during in vitro renaturation steps.

### Western Blot Analysis of VprSJ4

**Fig. 4:**
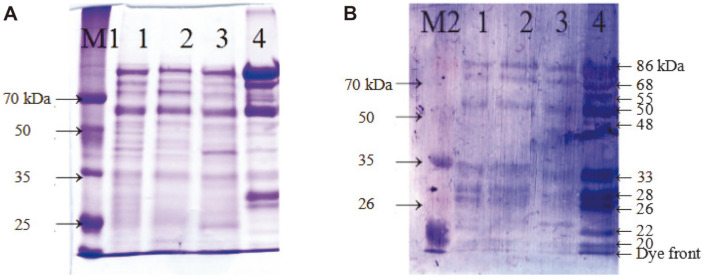
SDS-PAGE and western blot of VprSJ4 purified from insoluble fraction. (**A**) SDS-PAGE of purified VprSJ4 using a HiTrap IMAC FF column. (**B**) Western blot analysis of VprSJ4. M1, Dokdo-marker (EBM-1034); M2, Dokdo-marker (prestained, EBM-1032); 1, insoluble fraction from *E. coli* BL21(DE3) [pETvprSJ4] grown for 20 h after IPTG induction; 2, flow through; 3, eluent from column flushed with wash buffer (20 mM sodium phosphate, pH 7.4, 20 mM imidazole, and 0.5 M NaCl); 4, eluent from column flushed with elution buffer with 100 mM imidazole concentration. After color development of the PVDF membrane with alkaline phosphatase substrate solution, the bands were purple. The color tone of the scan result was adjusted to blue to enhance the contrast.

Small fragments were detected by SDS-PAGE and western blot (Fig. 4), indicating autoprocessing of preproVprSJ4. Antibody against His-tag detected an 86 kDa band together with proteins of 68 kDa and smaller (55, 50, 48, 32, 28, 26, 22, and 20 kDa) (Fig. 4B). These small proteins might be degradation products of the 68 kDa mature Vpr by N-terminal processing since the His-tag was attached to C-terminus of Vpr. In a previous study on Vpr from *B. subtilis* 168, small proteins detected by His-tag antibody were identified as N-terminus deletion derivatives of Vpr by MALDI-TOP mass spectrometry [15]. In another study, Vpr was purified from culture supernatant by gel filteration and eluted from column in void volume, forming a complex with other proteins [21]. When the Vpr complex was subjected to SDS-PAGE, 38, 28.5, and 27 kDa bands were separated. When N-terminal amino acids were determined, the 28 kDa protein had the same N-terminal amino acid sequence with the mature 68 kDa Vpr, indicating C-terminal processing of Vpr. For 27 kDa protein, the first 9 amino acids of mature enzyme were missing [21]. These reports indicated that both N-terminal and C-terminal processing of Vpr occur in vivo.

A few bands were observed between 20 and 32 kDa (lane 4, Fig. 4B), and 26 and 28 kDa bands were likely processed derivatives of Vpr. Further study will be necessary to confirm the exact processing of VprSJ4 in vivo.

### Properties of Recombinant VprSJ4

**Fig. 5:**
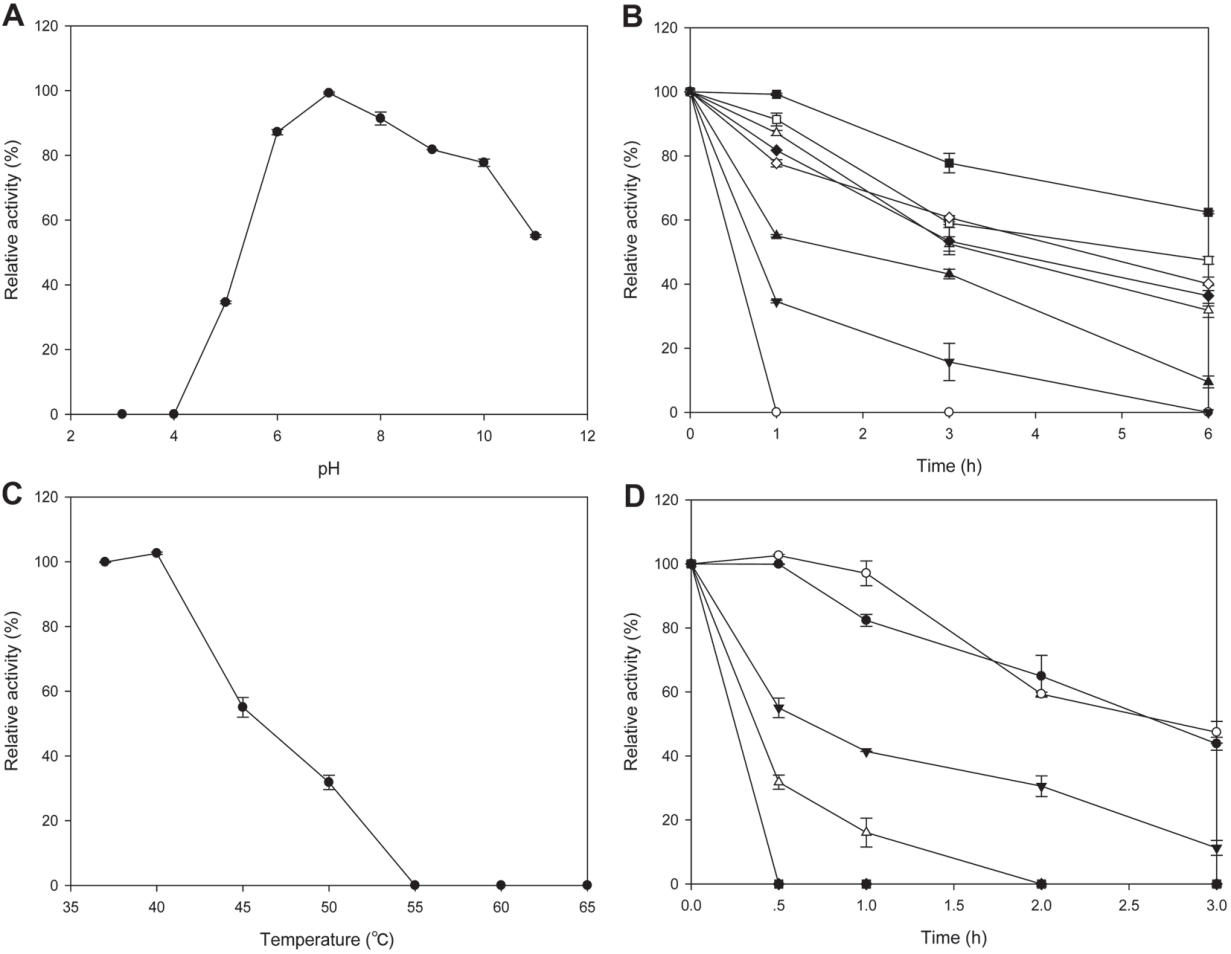
Properties of Recombinant VprSJ4. VprSJ4 eluted at 200 mM imidazole concentration was centrifuged using an Ultra-15 Centrifugal Filter (Amicon, 3 kDa, MWCO) to remove imidazole. Desalted VprSJ4 was used for studies on its properties. pH 7.0 was the optimum pH for the activity and VprSJ4 maintained higher activity at pH 6-10 (Fig. 5A). The optimum pH was 9.0 for partially purified Vpr from *B. subtilis* KCTC3014 [22]. The difference in optimum pH might be due to the different amino acid sequences or additional 4 amino acids of VprSJ4 at the C-terminus. VprSJ4 showed relative activity of 87.2, 91.4, 81.7, and 77.7% at pH 6, 8, 9, and 10, respectively. VprSJ4 showed no activity at pH 4 and below. The activity declined rapidly at pH above 10. During 6 h incubation, the activity decreased continuously at all pHs, and rapid inactivation occurred at pH 5 and below (Fig. 5B). The optimum temperature was 40°C at pH 7 (Fig. 5C), which was the same temperature reported for partially purified Vpr from *B. subtilis* KCTC3014 [22]. When the temperature was increased to 55°C and above, no activity was detected after 30 min incubation. During 3 h incubation, the activity decreased continuously, and especially decreased rapidly at 50°C and above. The results showed that VprSJ4 has moderate stabilities against pH and temperature.

VprSJ4 eluted at 200 mM imidazole concentration was centrifuged using an Ultra-15 Centrifugal Filter (Amicon, 3 kDa, MWCO) to remove imidazole. Desalted VprSJ4 was used for studies on its properties. VprSJ4 maintained higher activity at pH 6-10, and the highest activity was observed at pH 7.0 (Fig. 5A), which was lower (pH 9.0) than that of partially purified Vpr from *B. subtilis* KCTC3014 [22]. The difference in optimum pH might be due to the difference in amino acid sequence or additional 4 amino acids at the C-terminus. Relative activity of 87.2, 91.4, 81.7, and 77.7% were observed at pH 6, 8, 9, and 10, respectively. The enzyme had no activity at pH 4 and below. The activity declined rapidly at pH above 10; it continued to decrease over 6 h incubation at all pHs, and rapid inactivation occurred at pH 5 and below (Fig. 5B).

The optimum temperature was 40°C at pH 7 (Fig. 5C), which was the same as that of partially purified Vpr from *B. subtilis* KCTC3014 [22]. When we increased the temperature to 55°C and above, no activity remained after 30 min incubation. The activity decreased continuously over 3 h incubation, and also decreased rapidly at 50°C and above. The results showed that VprSJ4 has moderate stabilities against pH and temperature.

The effects of metals and inhibitors on the activity of VprSJ4 are presented in Table 1. Ca^2+^ enhanced the fibrinolytic activity by 8.74% followed by Mg^2+^ (5.60%) and Mn^2+^ (2.06%). The activity was inhibited by Co^2+^ (13.28% inhibition) and Zn^2+^ (13.28% inhibition). The fibrinolytic activity was completely inhibited by PMSF, and most of the activity was inhibited by EDTA (84.87% inhibition), and EGTA (77.01% inhibition), but was not affected by SDS. The results showed that VprSJ4 is a serine protease and also a metal protease.

### Kinetics and Amidolytic Activity Measurements

The *K*_m_ and *V*_max_ of recombinant VprSJ4 were 0.19 mM and 29.76 μM/min, respectively, and *K*_cat_ was 33.96 S^-1^. *K*_cat_/*K*_m_ of recombinant VprSJ4 was 1.79 × 10^5^ S^-1^ M^-1^. As far as we know, this is the first report on the kinetic parameters of Vpr from *Bacillus* species. The values were compared with those of other fibrinolytic enzymes from *Bacillus* species where kinetic parameters were reported by using the same substrate, *N*-succinyl-ala-ala-pro-phe-*p*-nitroanilide. *B. pumilus* BS15 was isolated from jeotgal made with oyster and its main fibrinolytic enzyme, AprEBS15, showed *K*_m_ and *V*_max_ of 0.26 mM and 21.88 μM/min, respectively [23]. The *K*_cat_ was 10.02 S^-1^, and *K*_cat_/*K*_m_ was 3.85 × 10^4^ S^-1^ M^-1^. *B. subtilis* HK176 was isolated from cheonggukjang and its main fibrinolytic enzyme gene, *aprE176*, was cloned [19]. The *K*_m_, *K*_cat_, and *K*_cat_/*K*_m_ of AprE176 were 0.453 mM, 122.851 S^-1^, and 2. 71 × 10^5^ S^-1^ M^-1^, respectively [19]. *B. subtilis* DC33, an isolate from the Chinese fermented food douchi (salted black soybean), secretes a 30 kDa subtilisin-type enzyme, which showed *K*_m_, *K*_cat_, and *K*_cat_/*K*_m_ of 0.21 mM, 37.04 S^-1^, and 1.76 ×10^5^ S^-1^ M^-1^, respectively [24]. *B. subtilis* IMR-NK1, a strain used for natto production, produces a 31.5 kDa fibrinolytic enzyme, and the enzyme showed *K*_m_, *K*_cat_, and *K*_cat_/*K*_m_ of 0.34 mM, 21.08 S^-1^, and 6.2 × 10^4^ S^-1^ M^-1^, respectively [25]. Another fibrinolytic enzyme from a *B. subtilis* strain used for red bean natto production showed *K*_m_, *K*_cat_, and *K*_cat_/*K*_m_ of 0.59 mM, 39.6 S^-1^, and 6.7 × 10^4^ S^-1^ M^-1^ , respectively [26]. *Bacillus* sp. nov. SK006 was isolated from fermented shrimp paste and a fibrinolytic enzyme, 43-46 kDa in size, was purified, and showed *K*_m_, *K*_cat_, and *K*_cat_/*K*_m_ of 0.45 mM, 28.75 S^-1^, and 6.4 × 10^4^ S^-1^ M^-1^, respectively [27]. VprSJ4 has a lower *K*_m_ value than other fibrinolytic enzymes, mainly AprE type, indicating higher affinity for the substrate. VprSJ4 shows similar or slightly higher catalytic efficiency (*K*_cat_/*K*_m_ value) than other enzymes except AprEHK176. AprEHK176 has slightly higher efficiency than VprSJ4.

### Coexpression of *aprESJ4* and *vprSJ4* in *B. subtilis* WB600

**Fig. 6:**
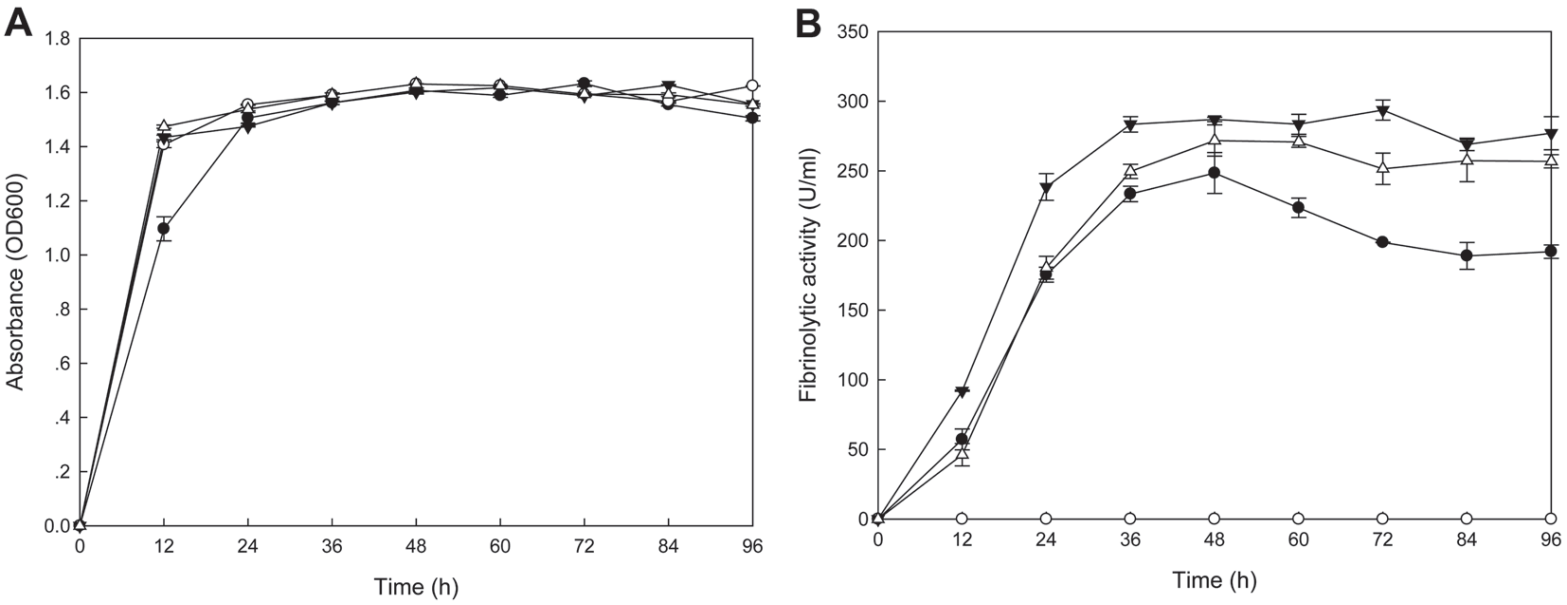
Growth (A) and fibrinolytic activities (B) of *B. subtilis* WB600 strains. -●-, *B. subtilis* SJ4; -○-, *B. subtilis* WB600 [pHY300PLK]; -▼-, *B. subtilis* WB600 [pHYavSJ4]; -△-, *B. subtilis* WB600 [pHYaprESJ4].

*B. subtilis* WB600 harboring pHYavSJ4 (pHY300PLK containing *aprESJ4* and vprSJ4) showed the same growth curve as *B. subtilis* WB600 [pHY300PLK] (control) and *B. subtilis* SJ4 (Fig. 6A). *B. subtilis* WB600 [pHYavSJ4] showed the highest fibrinolytic activity (293.6 U/ml) at 72 h whereas control did not show any significant activity (Fig. 6B). *B. subtilis* SJ4 showed the highest fibrinolytic activity (248.4 U/ml) at 48 h and *B. subtilis* WB600 [pHYaprE34] showed the highest activity (271.8 U/ml) at 48 h. *B. subtilis* WB600 [pHYaprESJ4] showed higher fibrinolytic activity than *B. subtilis* SJ4, but lower than *B. subtilis* WB600 [pHYavSJ4]. At 72 h, the activity of *B. subtilis* WB600 [pHYavSJ4] was 1.48 fold and 1.17 fold higher than that of *B. subtilis* SJ4 and *B. subtilis* WB600 [pHYaprESJ4], respectively. The result indicated that coexpression of *vprSJ4* and *aprESJ4* caused additional increase in the fibrinolytic activity of *B. subtilis* WB600. The activity could be increased further if efforts to optimize gene expression of *aprESJ4* and *vprSJ4* are made in the future, along with the use of other vectors and promoter changes.

**Fig. 7:**
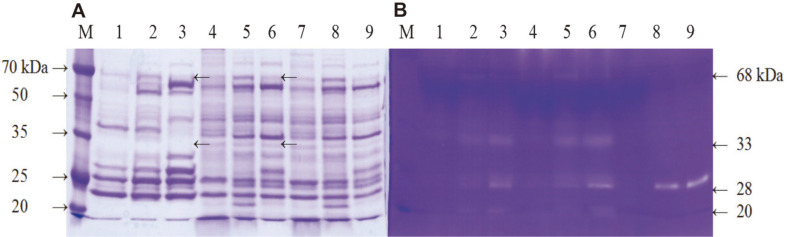
Coomassie Blue-stained gel (A) and fibrin zymogram (B) of culture supernatant from *B. subtilis* WB600 strains. M, Dokdo-marker (EBM-1034); lanes 1-3, *B. subtilis* SJ4 culture supernatant at 24 h (1), 48 h (2), and 96 h (3); lanes 4-6, *B. subtilis* WB600 [pHYavSJ4] culture supernatant at 24 h (4), 48 h (5), and 96 h (6); lanes 7-9, *B. subtilis* WB600 [pHYaprESJ4] culture supernatant at 24 h (7), 48 h (8), and 96 h (9).

SDS-PAGE of culture supernatant of *B. subtilis* WB600 [pHYavSJ4] showed that sizes of 68, 60, 45, 33, 28, 25, and 22 kDa were observed in addition to other minor bands on a Coomassie Blue-stained gel (Fig. 7A). The zymogram (Fig. 7B) of the recombinant strain (lanes 4-6) clearly showed the mature VprSJ4 band (68 kDa) and the mature AprESJ4 band (28 kDa). The control (lanes 7-9) showed only the mature AprESJ4 band. The 33k Da band observed on the zymogram (lanes 4-6) was a degradation product from VprSJ4. The overall profiles of the zymogram are the same for *B. subtilis* SJ4 and *B. subtilis* WB600 [pHYavSJ4]. The zymogram results indicated that AprESJ4 as well as VprSJ4 are largely responsible for the fibrinolytic activity of *B. subtilis* SJ4, and VprSJ4 seemed to be autoprocessed in a heterologous host, just as in the original host. *B. subtilis* WB600 seemed to be deficient in Vpr activity since 68 and 33 kDa bands were not observed in cells carrying only *aprESJ4* (Fig. 7B, lanes 7-9).

*B. subtilis* SJ4 has good potential for use as a starter for fermented foods, especially foods containing less than 15% salt (w/v), because of its GRAS status, strong fibrinolytic activity, and significant salt tolerance. The fibrinolytic activity of *B. subtilis* SJ4 can enhance the functionality of fermented foods where the strain is used as a starter. Fibrinolytic enzymes from *Bacillus* species have potential to be developed as an alternative to t-PA, urokinase, or streptokinase, which are prescribed for preventing or treating cardiovascular diseases by dissolving fibrin clots in blood vessels [4, 6]. Unlike the case of AprE, not many studies have been done on Vpr, neither on its roles during growth at later exponential phase and stationary phase, nor its contribution to the overall fibrinolytic capacity of a *Bacillus* strain. Further studies on Vpr and other minor proteases will help us understand the fibrinolytic capacity of *Bacillus* species and find ways to utilize these fibrinolytic enzymes for medicinal uses and production of functional foods.

## Supplemental Materials



Supplementary data for this paper are available on-line only at http://jmb.or.kr.

## Figures and Tables

**Table 1:** Effects of metal ions and inhibitors on the activity of VprSJ4.

Metal ions (5 mM)	Relative activity (%)	Inhibitors (1 mM)	Relative activity (%)
None	100	PMSF	0.00±0.00
Mn^2+^	102.06±0.56	EDTA	15.13±0.92
Mg^2+^	105.60±0.59	EGTA	22.99±2.60
Ca^2+^	108.74±0.59	SDS	81.38±2.41
K^+^	100.93±0.97		
Zn^2+^	86.72±1.05		
Fe^3+^	92.21±0.06		
Na^+^	99.61±0.19		
Co^2+^	86.72±1.05		

The counter ion for the tested metals was chloride.

All values are the mean ± SD (*n* = 3).
