# Supplementary material for: Cloning of a Novel vpr Gene Encoding a Minor Fibrinolytic Enzyme from Bacillus subtilis SJ4 and the Properties of Vpr
Source: J Microbiol Biotechnol. 2020 Aug 21;30(11):1720–8. doi: 10.4014/jmb.2006.06014 (PMC9728201; doi:10.4014/jmb.2006.06014)
Supplement: Supplementary file 1 [file JMB-30-11-1720-supple.pdf]

J. Microbiol. Biotechnol.
https://doi.org/10.4014/jmb.2006.06014

```
M K K G I I R F L L V S F V L F F T L S T G I T G V K A▼A P
TTGAAAAAGGGGATCATTCGCTTTCTGCTTGTCAGTTTCGTCTTATTTTTTACGTTATCCACAGGTATTACGGGCGTCAAGGCCGCTCCG   90
V S S K T S A D L E K A E V F G D I D M T T S K K T T V I V
GTATCTTCAAAAACGTCGGCTGACCTGGAAAAAGCCGAGGTATTCGGTGATATCGACATGACAACAAGCAAAAAAACAACCGTTATTGTG  180
E L K E K S L A E A K E A G E S Q S K S K L K T A R T K A K
GAATTAAAAGAAAAATCCTTGGCAGAAGCGAAGGAAGCGGGAGAAAGCCAATCGAAAAGCAAGCTGAAAACCGCTCGCACCAAAGCAAAA  270
N K A I K A V K N G K V N R E Y E Q V F S G F S M K L P A N
AACAAAGCAATCAAAGCGGTGAAAAACGGAAAAGTAAACCGGGAATATGAGCAGGTATTCTCAGGCTTCTCTATGAAGCTTCCAGCTAAT  360
E I P K L L T V K D V K A V Y P N V T Y K T D N M K D K D V
GAGATTCCAAAACTTCTCACGGTAAAAGACGTTAAGGCAGTGTACCCGAACGTCACATATAAAACAGACAATATGAAGGATAAAGACGTC  450
T I S E D A V S P Q▽M D D S A P Y I G A N D A W D L G Y T G
ACAATCTCCGAAGACGCCGTATCTCCGCAAATGGATGACAGTGCGCCTTATATCGGAGCAAACGATGCATGGGATTTAGGCTACACAGGA  540
K G I K V A I I D T G V E Y N H P D L K K N F G Q Y K G H D
AAAGGCATCAAGGTGGCGATTATTGACACTGGGGTTGAATACAATCACCCAGATCTGAAGAAAAACTTTGGACAATATAAAGGACACGAT  630
F V D N D Y D P K E T P T G D P R G G A T D H G T H V A G T
TTTGTGGACAATGATTACGATCCAAAAGAAACACCAACAGGCGATCCGAGGGGCGGAGCGACTGACCACGGCACACACGTAGCCGGAACT  720
V A A N G T I K G V A P D A T L L A Y R V L G P G G S G T T
GTGGCTGCAAACGGCACAATTAAAGGGGTAGCGCCTGATGCCACGCTTCTTGCTTATCGTGTGTTAGGACCTGGCGGAAGCGGCACAACG  810
E N V I A G V E R A V Q D G A D V M N L S L G N S L N N P D
GAAAACGTCATCGCGGGCGTGGAACGCGCTGTACAGGACGGGGCAGATGTGATGAACCTGTCTCTCGGAAACTCTTTAAACAACCCGGAC  900
W A T S T A L D W A M S E G V V A V T S N G N S G P N G W T
TGGGCGACAAGCACAGCGCTTGACTGGGCCATGTCAGAAGGCGTTGTCGCTGTTACCTCAAACGGCAACAGCGGACCGAACGGCTGGACA  990
V G S P G T S R E A I S V G A T Q L P L N E Y A V K F G S Y
GTCGGATCGCCGGGCACATCAAGAGAAGCGATTTCTGTCGGTGCGACTCAGCTGCCGCTCAATGAATACGCCGTAAAATTCGGTTCCTAT 1080
S S A K V M G Y N K E D D V K A L N N K E V E L V E A G I G
TCTTCAGCAAAAGTGATGGGCTATAACAAGGAGGACGACGTCAAAGCGCTCAATAATAAAGAAGTTGAGCTTGTCGAAGCGGGAATCGGC 1170
E A K D F E G K D L T G K V A V V K R G S I A F V D K A D N
GAAGCAAAGGATTTTGAAGGGAAAGACCTGACAGGCAAAGTCGCCGTTGTCAAACGAGGCAGCATTGCATTTGTGGATAAAGCGGATAAC 1260
A K K A G A I G M V V Y N N L S G E I E A N V P G M S V P T
GCTAAAAAAGCCGGTGCCATCGGCATGGTTGTGTATAACAACCTCTCTGGAGAAATTGAAGCCAATGTGCCAGGCATGTCTGTCCCAACG 1350
I K L S L E D G E K L V S A L K A G E T K T T F K L T V S K
ATTAAGCTTTCATTAGAGGACGGCGAAAAACTAGTCAGCGCCCTGAAAGCTGGTGAGACAAAAACAACATTCAAGTTGACGGTCTCAAAA 1440
A L G E Q V A D F S S R G P V M D T W M I K P D I S A P G A
GCGCTCGGTGAACAAGTGGCTGATTTCTCATCACGCGGCCCTGTTATGGATACGTGGATGATTAAGCCTGATATTTCGGCGCCAGGGGCC 1530
N I V S T I P T H D P G H P Y G Y G S K Q G T S M A S P H I
AATATCGTCAGCACGATCCCAACACACGATCCTGGCCATCCATACGGCTACGGATCAAAACAAGGAACAAGCATGGCATCGCCTCATATT 1620
A G A V A V I K Q A K P K W S V E Q I K A A I M N T A V T L
GCCGGAGCGGTTGCCGTTATTAAACAAGCCAAACCAAAGTGGAGCGTTGAACAGATTAAAGCCGCCATCATGAATACCGCTGTCACTTTA 1710
K D G D G E V Y P H N A Q G A G S A R I M N A I K A D S L V
AAGGATGGCGATGGGGAAGTATATCCGCATAACGCTCAAGGCGCGGGCAGCGCAAGAATTATGAACGCGATCAAAGCCGATTCGCTCGTC 1800
S P G S Y S Y G T F L K E N G N E T K N E T F T I E N Q S S
TCACCTGGAAGCTATTCATACGGCACATTCTTGAAGGAAAACGGAAACGAAACGAAAAATGAAACGTTTACGATTGAAAATCAATCTTCC 1890
I R K S Y T L E Y S F N G S G I S T S G T S R V V I P A H Q
ATTAGAAAATCATACACACTGGAATACTCATTTAATGGCAGCGGCATCTCCACATCCGGCACAAGCCGTGTTGTGATTCCGGCACATCAA 1980
T G K A T A K V K V N T K K T K A G T Y E G T V I V R E G G
ACCGGGAAAGCCACTGCAAAAGTAAAGGTCAATACGAAGAAAACAAAAGCTGGCACCTATGAAGGAACGGTTATCGTCAGAGAAGGCGGA 2070
K T V A K V P T L L I V K E P D Y P R V T S V S V S E G S V
AAAACGGTCGCTAAGGTACCTACATTGCTGATTGTGAAAGAGCCCGATTATCCGAGAGTCACATCTGTCTCCGTAAGCGAAGGGTCTGTA 2160
Q G T Y Q I E T Y L P A G S E E L A F L V Y D S N L D F A G
CAAGGCACCTATCAAATTGAAACCTACCTTCCTGCGGGATCGGAAGAGCTGGCGTTTCTCGTCTATGACAGCAACCTTGATTTCGCAGGC 2250
Q A G I Y K N Q D K G Y Q Y F D W D G T I N G G T K L P A G
CAAGCCGGCATTTATAAAAATCAAGATAAAGGTTATCAGTACTTTGACTGGGACGGCACGATTAATGGCGGAACCAAGCTTCCGGCCGGA 2340
E Y Y L L A Y A A N K G K S S Q V L S E E P F T V E D E F A
GAGTATTACTTGCTCGCATATGCCGCGAACAAAGGCAAGTCAAGCCAGGTTTTGTCCGAAGAACCTTTCACTGTTGAAGACGAATTCGCG 2430
-
TAA  2433
```

Suppl. Fig. 1. Nucleotide sequence of *vprSJ4*.

The deduced amino acid sequence is shown below the nucleotide sequence. The ends of the pre (▼) and pro sequences (▽) are marked.

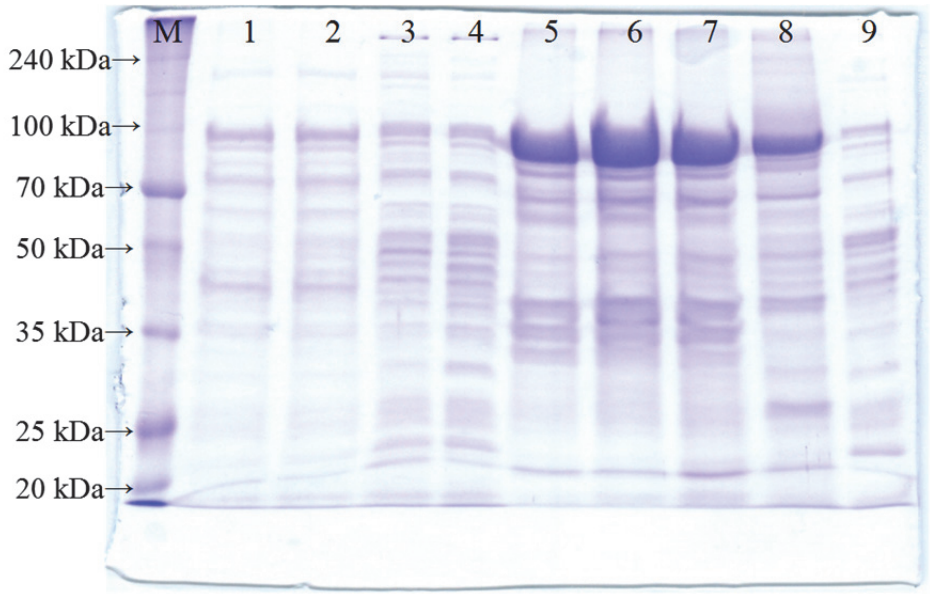

Suppl. Fig. 2. Overexpression of *vprSJ4* in *E. coli*.

M, Dokdo-marker (EBM-1034, Elpis Biotech, Korea);

Lanes 1-4, soluble fraction from *E. coli* BL21 [pETvprSJ4] cells grown for 2 h (1), 4 h (2), 10 h (3), and 20 h (4) after IPTG induction; lanes 5-8, insoluble fraction from *E. coli* BL21 [pETvprSJ4] cells grown for 2 h (5), 4 h (6), 10 h (7), and 20 h (8) after IPTG induction; 9, insoluble fraction from *E. coli* BL21 [pET26b(+)] grown for 20 h after induction (negative control).
